# CONSORT Item Reporting Quality in the Top Ten Ranked Journals of Critical Care Medicine in 2011: A Retrospective Analysis

**DOI:** 10.1371/journal.pone.0128061

**Published:** 2015-05-28

**Authors:** Ana Stevanovic, Sabine Schmitz, Rolf Rossaint, Tobias Schürholz, Mark Coburn

**Affiliations:** 1 Department of Anaesthesiology, University Hospital Aachen, Aachen, Germany; 2 Department of Intensive Care and Intermediate Care, University Hospital Aachen, Aachen, Germany; Carl von Ossietzky University of Oldenburg, GERMANY

## Abstract

**Introduction:**

Reporting randomised controlled trials is a key element in order to disseminate research findings. The CONSORT statement was introduced to improve the reporting quality. We assessed the adherence to the CONSORT statement of randomised controlled trials published 2011 in the top ten ranked journals of critical care medicine (ISI Web of Knowledge 2011, Thomson Reuters, London UK).

**Methods:**

**Design.** We performed a retrospective cross sectional data analysis. **Setting.** This study was executed at the University Hospital of RWTH, Aachen. **Participants.** We selected the following top ten listed journals according to ISI Web of Knowledge (Thomson Reuters, London, UK) critical care medicine ranking in the year 2011: American Journal of Respiratory and Critical Care Medicine, Critical Care Medicine, Intensive Care Medicine, CHEST, Critical Care, Journal of Neurotrauma, Resuscitation, Pediatric Critical Care Medicine, Shock and Minerva Anestesiologica. **Main outcome measures.** We screened the online table of contents of each included journal, to identify the randomised controlled trials. The adherence to the items of the CONSORT Checklist in each trial was evaluated. Additionally we correlated the citation frequency of the articles and the impact factor of the respective journal with the amount of reported items per trial.

**Results:**

We analysed 119 randomised controlled trials and found, 15 years after the implementation of the CONSORT statement, that a median of 61,1% of the checklist-items were reported. Only 55.5% of the articles were identified as randomised trials in their titles. The citation frequency of the trials correlated significantly (rs = 0,433; p<0,001 and r = 0,331; p<0,001) to the CONSORT statement adherence. The impact factor showed also a significant correlation to the CONSORT adherence (r = 0,386; p<0,001).

**Conclusion:**

The reporting quality of randomised controlled trials in the field of critical care medicine remains poor and needs considerable improvement.

## Introduction

### Background

Randomised controlled trials (RCTs) are known to provide the best quality research evidence [1,2]. Therefore RCTs should evince the best possible quality of methodology [2,3]. Qualitative reporting is closely linked with methodological quality [3] and poor reporting leads to an overestimation of the trial effect [4,5]. The CONSORT (Consolidated Standards of Reporting Trials) statement was developed (first 1998, revised 2001 and 2010) [6–8] to maximize the reporting quality of RCTs and increase the transparency of the quality of findings to the readers. The CONSORT statement enables structured reporting of RCTs, simplifies comparisons of the trials and reduces bias [9]. Since its implementation, several studies have investigated the effect of the CONSORT statement on the quality of new published RCTs in specific medical disciplines [10–13]. They recommend the obligatory use of the CONSORT statement when designing the RCTs, pertinent to the submissions procedures of journals [2,14].

### Objectives

Our aim was to analyse the reporting quality of RCTs in the top ten ranked journals in the field of critical care medicine (via ISI Web of Knowledge 2011, Thomson Reuters, London, UK) according to the CONSORT statement published in 2010 [9]. The potential surrogate marker for the quality of publications, like paper citation frequency and the impact factor of the journal, was correlated to the adherence of each RCT to the CONSORT statement.

## Methods

### Study design

We performed a retrospective cross sectional analysis of data published in the entire year of 2011 and reported the data according to the STROBE statement (S1 Checklist) [15].

### Setting

This analysis was conducted at the University Hospital of RWTH Aachen.

### Unit of analyses

**Fig 1:**
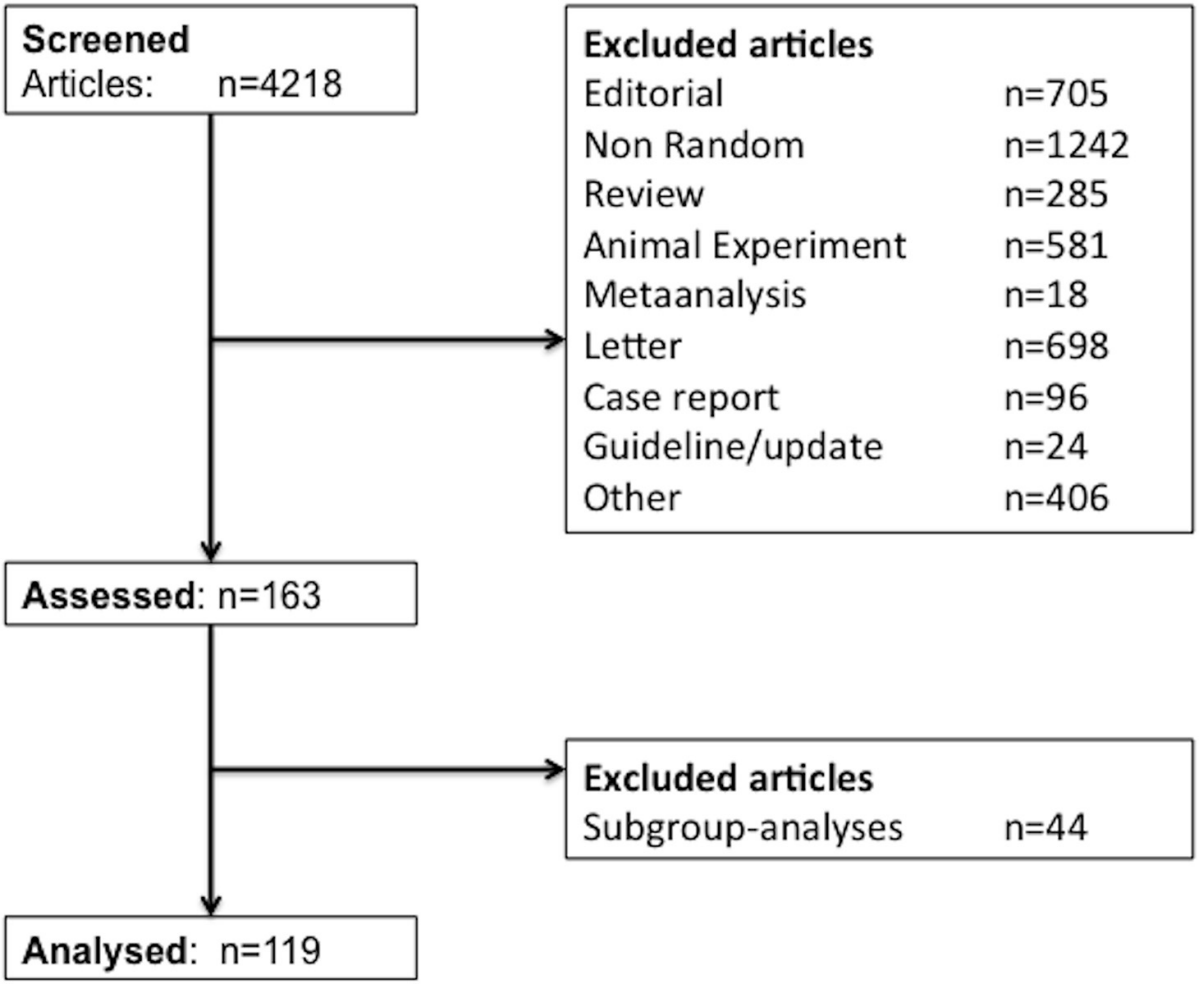
Flowchart. Flowchart showing the screening and inclusion process for randomised controlled trials, which were included or excluded in the current study.

We selected the listed top ten ranked journals in the category of critical care medicine according to their impact factor in 2011 (identified via ISI Web of Knowledge, journal citation reports). One author (SS) screened all articles published in 2011 in the aforementioned journals, to identify RCTs. We excluded by screening the titles and abstracts, all other types of publications (Fig 1). Discrepancies regarding the study allocation were discussed with a second author (MC) and in the event of remaining discrepancies a further author (AS) was involved. All primary reports of prospective randomised controlled trials in human participants were included.

### Data extraction and variable definition

To minimize subjective interpretations, clear default categories of the CONSORT items were established (SS and MC). A data sheet was drafted, which implemented these defaults and the CONSORT checklist and elaboration document. Every RCT was analysed by screening the full text and all supplements (SS) to identify each of the 37 CONSORT items, thus every RCT could achieve a maximum score of 37. Every item was marked, with “positive”, if reported, and if not, “negative”. A further option “not applicable” applied to item 11b “similarity of interventions”, since not all trials were blinded. We only analysed the quantity of reported items, we did not analyse the quality of the reported content. The decision defaults for allocation of each item are shown in S1 Table. The citation frequency of every included RCT article was assessed, in the period from 01.01.2012–31.12.2014, on the ISI Web of Knowledge website for correlation analyses.

### Bias

To minimize selection bias, we screened the online table of content directly on each journals`website and not only online databases. Every uncertainty arising from the correct assignment of the CONSORT items to the included RCTs was clarified consistently with the other authors in the full text (MC, AS). Furthermore, a second author (AS) crosschecked a random sample of 12 RCTs (10% of all included RCTs), to validate an unambiguous allocation of the checklist items. The inter-rater reliability was assessed by the kappa statistic for these random samples. Blinding to the authors`and journals`names during the assessment process was not performed, due to practicability and the lack of evidence for this method to exclude bias [16].

### Statistical Methods

We performed all our statistical analyses using SPSS 21 Statistics Software (IBM Corporation, Armonk, NY, USA). At the article level we computed the percentage of all RCTs reporting each CONSORT item and the percentage adherence of each RCT to all CONSORT items. For the summary statistic, we computed the mean and standard deviation, the median and range and the Huber M-estimate with Huber weights as the adherence proportion showed long tails [17]. At the journal level we assigned all RCTs to the respective journals and calculated the percentage of reporting each item for the respective journals. Additionally the previously described summary statistic was also calculated for the total percentage-reporting adherence, of all journals to each item. All CONSORT items were weighted equally. Three categories of fulfilling the checklist items (below 50%, between 50% and 80% and above 80%) have been created to distinguish between the respective journals`adherence. The distribution of RCTs depending on the proportion of reported items (%) was derived using the Gaussian kernel density estimation [18]. Finally, we assessed whether there is any correlation, at the article level, between the adherence to the CONSORT items and the number of citations from 01.01.2012–31.12.2014. For these correlation studies we have used the Spearman`s rank correlation (computing the coefficient r_s_) and the Pearson`s correlation analysis (computing the coefficient r). A p-value of <0.05 was set to be significant. The correlation analysis of the respective journal`s impact factor in 2011 and the adherence to the CONSORT items was performed by using the Spearman`s rank correlation.

## Results

### Articles

We identified a total of 4218 publications directly on the table of contents of each included “top ten” journal, for the entire year 2011. Through a manually screening process of titles and abstracts (SS), we excluded 4055 publications, as they were not RCTs. Additional 44 publications were excluded, as they were only subgroup analyses and not primary studies. The remaining 119 studies were identified as RCTs and were included in our analysis (Fig 1).

### Main results

**Fig 2:**
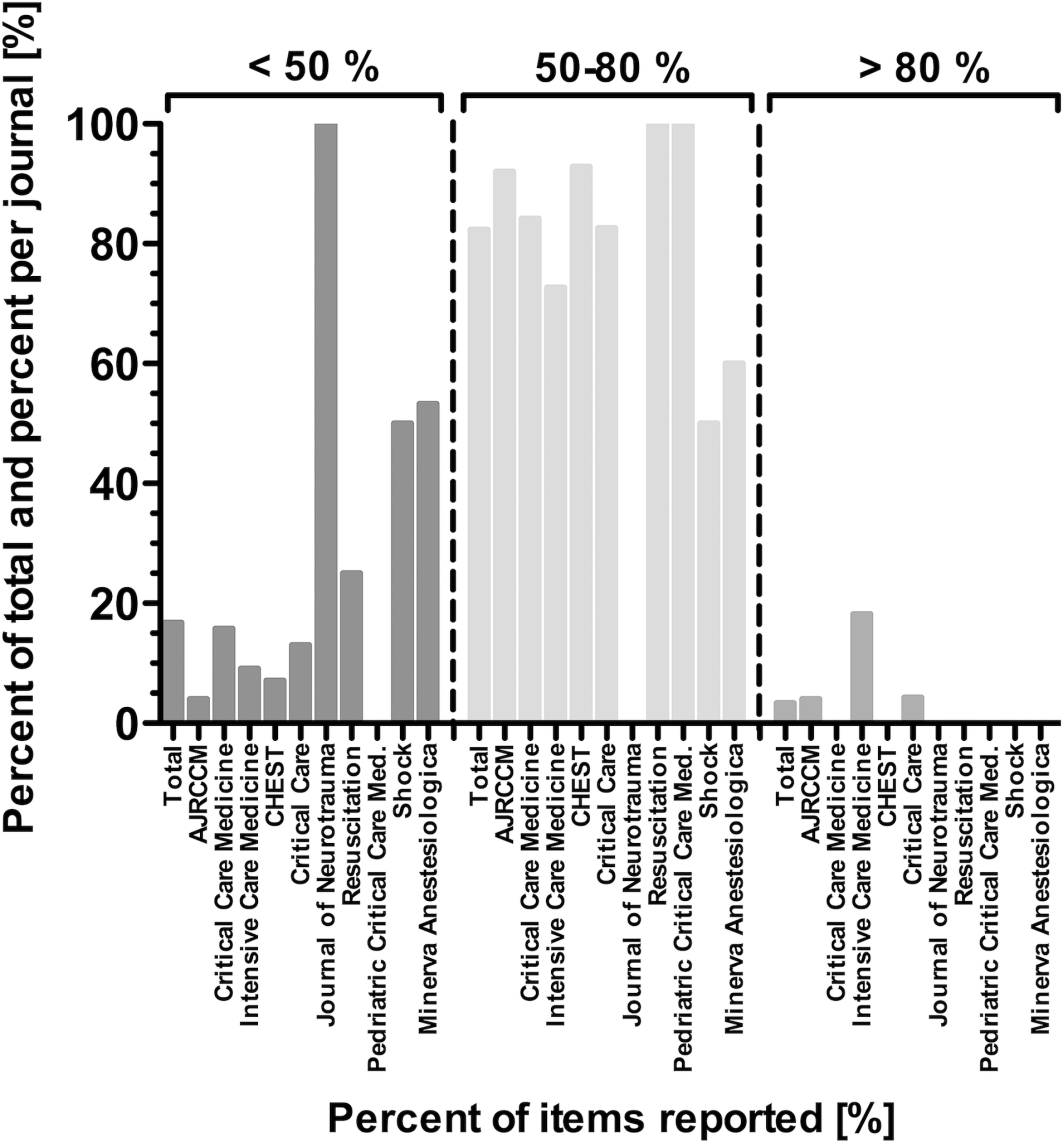
Overview of journals`adherence to CONSORT checklist items. Shown are three categories of adherence to the 37 respective 36 (in case of not applicability of item 11b) CONSORT checklist items: below 50%, between 50% and 80%, above 80%. Results are shown in percent per journal and of all assessed journals (total).

**Fig 3:**
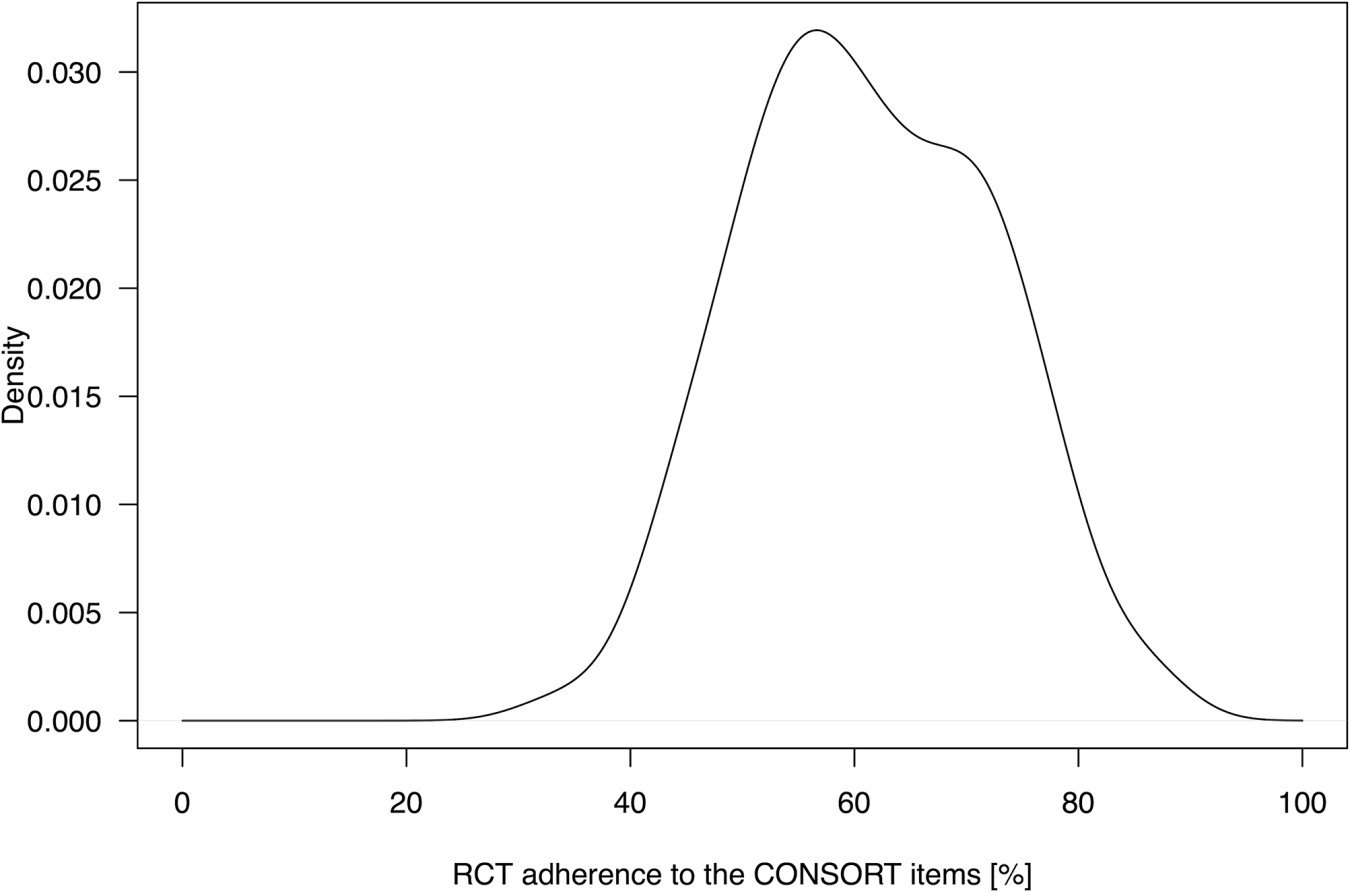
Adherence of RCTs to CONSORT checklist. Kernel density estimation showing the percentage of the 37 respective 36 (in case of not applicability of item 11b) possible CONSORT items that were reported in 119 randomised controlled trials.

The percentage adherence of all 119 RCTs, published 2011 in the top-ten critical care medicine journals, to each CONSORT item respective the total of items is shown in Table 1. Furthermore we show the summary statistic for the adherence of each RCT to all CONSORT items in Table 1. The included RCTs reported in a median of 61,1% of all required CONSORT checklist items with a range of 33,3–86,5%. The standard robust estimator, Huber`s M-estimate, showed the same result with 61,1% and precludes a potential skewness of the adherence in the checklist. The percentages of trials belonging to one journal and reporting each CONSORT item are shown in Table 2. The total reporting adherence of all journals to each CONSORT item is additionally shown in Table 2. The total percentage of reporting adherence per journal was most frequent in the 50–80% range (Fig 2). Most articles reported 50–60% of the CONSORT items (Fig 3). Only two items (2a = scientific background and rationale and 22 = interpretation of the study results) were reported in every included RCT. Eight items were reported in more than 90% (Table 1): 1b = structured abstract, 2b = specific objectives/hypotheses, 4a = eligibility criteria for participants, 5 = detailed intervention description for each group, 6a = detailed description of primary and secondary outcome measures, 11b = similarity of interventions, 12a = statistical methods used to compare groups for primary and secondary outcomes, 17a = estimated effect size and its precision of primary and secondary outcomes for each group. Five items were reported in less than 10% (3b = method changes after trial onset, 6b = changes of trial outcomes after trial commencement, 12b = methods of additional analyses, 18 = Results of additional analyses and 24 = Where the study-protocol can be accessed).

**Table 1:** RCT adherence to CONSORT Checklist.

	Item description	Item n°	Adherence proportion of all 119 RCTs(%)
**Title & abstract**	Identification as a randomised trial in the title	**1a**	55,5
	Structured summary (design, methods, results, conclusions)	**1b**	97,5
**Introduction**	Scientific background and explanation of rationale	**2a**	100
	Specific objectives or hypotheses	**2b**	99,2
**Methods**			
**Trial design**	Description of trial design incl. allocation ratio	**3a**	84,9
	Changes to methods after trial commencement	**3b**	0
Participants	Eligibility criteria for participants	**4a**	99,2
	Settings and locations of collected data	**4b**	73,1
Interventions	Sufficient description of interventions for each group	**5**	98,3
Outcome	Pre-specified primary and secondary outcome measures	**6a**	96,6
	Changes to trial outcomes after trial start	**6b**	0,8
Sample size	How sample size was determined	**7a**	39,5
	Planned interim analyses and stopping guidelines	**7b**	10,1
Random sequence generation	How: Generation of random allocation sequence	**8a**	61,3
	Type of randomisation; details of any restriction	**8b**	50,4
Allocation concealment	How: Implementation of random allocation sequence and of sequence-concealment before assignment	**9**	37,0
Implementation	Who: generated the random allocation sequence, enrolled and assigned participants to interventions	**10**	19,3
Blinding	Who was blinded after assignment to interventions	**11a**	48,7
	Description of the similarity of interventions	**11b** ^**a**^	96,3^**a**^
Statistical methods	Statistical methods comparing primary and secondary outcomes	**12a**	98,3
	Methods for additional analyses (subgroup / adjusted)	**12b**	7,6
**Results**			
Participant Flow	Flowchart (or numbers randomised, received intended treatment, and analysed for the primary outcome)	**13a**	63,0
	Number and reasons for losses and exclusions	**13b**	69,7
Recruitment	Dates defining the periods of recruitment and follow-up	**14a**	72,3
	Why the trial ended or was stopped	**14b**	19,3
Baseline data	Table (baseline demographic and clinical characteristics)	**15**	89,1
Number analysed	Number of included participants in each analysis	**16**	83,2
Outcome & estimation	For each primary and secondary outcome, results for each group, and the estimated effect size and its precision	**17a**	95,0
	Absolute and relative effect sizes for binary outcomes	**17b**	47,9
Ancillary analyses	Results of other analyses (subgroup and adjusted analyses, distinguishing pre-specified from exploratory)	**18**	9,2
Harms	All important harms or unintended effects in each group	**19**	44,5
**Discussion**			
Limitations	Trial limitations (potential bias, imprecision)	**20**	82,4
Generalisability	Generalisability (external validity, applicability) of findings	**21**	75,6
Interpretation	Interpretation consistent with results, balancing benefits and harms, and considering other relevant evidence	**22**	100
**Other information**			
Registration	Registration number and name of trial registry	**23**	61,3
Protocol	Where the full trial protocol can be accessed	**24**	3,4
Funding	Sources of funding and other support, role of funders	**25**	87,4
**Summary statistic of the total percentage adherence to each applicable CONSORT item of all RCTs**			
**All items**	Mean and standard deviation (%)		61,5 ± 33,9
	Median and range (%)		69,7 [0–100]
	M-estimate (Huber) (%)		66,3
**Summary statistic of the total percentage adherence to all CONSORT items of each RCTs**			
**All items**	Mean and standard deviation (%)		61,2 ± 11,0
	Median and range (%)		61,1 [33,3–86,5]
	M-estimate (Huber) (%)		61,1

Table 1 shows the percentage adherence of all 119 RCTs to each CONSORT item. The summary calculation shows the values for each item reported by all RCTs and all items reported in each RCT.

^a^The percentage value calculation for item 11b^a^ is based on the number of RCTs for which this item was applicable, denominator: n = 82.

**Table 2:** Journal adherence to CONSORT Checklist.

	Item description	Item n°	Percentage adherence of alljournals median+[Range]	AJRCCM n = 25 (%)	Critical Care Medicine n = 19 (%)	Intensive Care Medicine n = 11 (%)	CHEST n = 14 (%)	Critical Care n = 23 (%)	Journal of Neurotrauma n = 1 (%)	Resuscitation n = 4(%)	Pediatric Critical Care Medicine n = 5 (%)	Shock n = 2 (%)	Minerva Anestesiologica n = 15 (%)
**Title & abstract**	Identification as a randomised trial in the title	**1a**	50 [0–71,4]	48,0	68,4	63,6	71,4	60,9	0	50,0	40,0	50,0	33,3
	Structured summary	**1b**	100 [0–100]	100	100	100	100	100	0	100	100	0	100
**Introduction**	Scientific background and explanation of rationale	**2a**	100[100–100]	100	100	100	100	100	100	100	100	100	100
	Specific objectives or hypotheses	**2b**	100[93,3–100]	100	100	100	100	100	100	100	100	100	93,3
**Methods**													
Trial design	Description of trial design incl. allocation ratio	**3a**	93,8[50–100]	72,0	94,7	90,9	92,9	95,7	100	50,0	100	100	66,7
	Changes to methods after trial commencement	**3b**	0	0	0	0	0	0	0	0	0	0	0
Participants	Eligibility criteria for participants	**4a**	100[94,7–100]	100	94,7	100	100	100	100	100	100	100	100
	Settings and locations of collected data	**4b**	76,4[42,9–100]	72,0	94,7	72,7	42,9	82,6	100	100	80,0	50,0	53,3
Interventions	Sufficient description of interventions for each group	**5**	100[94,7–100]	96,0	94,7	100	100	100	100	100	100	100	100
Outcome	Pre-specified primary and secondary outcome measures	**6a**	100 [50,0–100]	96,0	100	100	100	100	100	50,0	100	100	93,3
	Changes to trial outcomes after trial start	**6b**	0 [0–5,3]	0	5,3	0	0	0	0	0	0	0	0
Sample size	How sample size was determined	**7a**	41,8 [0–60,0]	40,0	26,3	45,5	35,7	43,5	0	0	60,0	50,0	53,3
	Planned interim analyses and stopping guidelines	**7b**	2,2 [0–36,4]	20,0	5,3	36,4	0	4,3	0	25,0	0	0	0
Random sequence generation	How: Generation of random allocation sequence	**8a**	59,3 [40–100]	64,0	68,4	54,5	42,9	69,6	100	50,0	40,0	50,0	66,7
	Type of randomisation; details of any restriction	**8b**	44,5 [25–100]	64,0	63,2	45,5	42,9	43,5	100	25,0	40,0	50,0	40,0
Allocation concealment	How: Implementation of random allocation sequence and of sequence-concealment before assignment	**9**	28,5 [0–52,6]	32,0	52,6	18,2	21,4	52,2	0	25,0	20,0	50,0	40,0
Implementation	Who: generated the random allocation sequence, enrolled and assigned participants to interventions	**10**	10,5[0–36]	36,0	15,8	27,3	14,3	21,7	0	0	0	0	6,7
Blinding	Who was blinded after assignment to interventions	**11a**	53,6[0–75,0]	48,0	21,1	63,6	57,1	60,9	0	75,0	60,0	50,0	40,0
	Description of the similarity of interventions	**11b**	100[83,3–100] n = 82	94,7 n = 19	94,7	100	100	100 n = 11	n.a.	100 n = 1	n.a.	100 n = 1	83,3 n = 6
Statistical methods	Statistical methods comparing primary and secondary outcomes	**12a**	100[92,9–100]	100	94,7	100	92,9	100	100	100	100	100	100
	Methods for additional analyses (subgroup / adjusted)	**12b**	0 [0–36,4]	12,0	10,5	36,4	0	0	0	0	0	0	0
**Results** Participant Flow	Flowchart (or numbers randomised, received intended treatment, and analysed for the primary outcome)	**13a**	62,3 [0–92,9]	76,0	68,4	63,6	92,9	60,9	0	75,0	40,0	0	26,7
	Number and reasons for losses and exclusions	**13b**	78,9 [0–100]	76,0	73,7	81,8	85,7	60,9	100	100	100	0	33,3
Recruitment	Dates defining the periods of recruitment and follow-up	**14a**	72,2 [0–90,9]	76,0	68,4	90,9	78,6	87,0	0	50,0	80,0	0	46,7
	Why the trial ended or was stopped	**14b**	0 [0–40,0]	40,0	10,5	27,3	0	34,8	0	0	0	0	0
Baseline data	Table (baseline demographic and clinical characteristics)	**15**	91,4 [0–100]	96,0	100	54,5	78,6	100	0	75,0	100	100	86,7
Number analysed	Number of included participants in each analysis	**16**	90,5 [50,0–100]	88,0	94,7	72,7	92,9	82,6	100	100	100	50,0	53,3
Outcome & estimation	For each primary and secondary outcome, results for each group, and the estimated effect size and its precision	**17a**	100[80,0–100]	100	94,7	100	92,9	95,7	100	100	100	100	80,0
	Absolute and relative effect sizes for binary outcomes	**17b**	44,2 [0–69,6]	52,0	63,2	45,5	42,9	69,6	0	50,0	20,0	0	13,3
Ancillary analyses	Results of other analyses (subgroup and adjusted analyses, distinguishing pre-specified from exploratory)	**18**	0 [0–50,0]	12,0	15,8	27,3	0	0	0	50,0	0	0	0
Harms	All important harms or unintended effects in each group	**19**	42,8[0–60,0]	56,0	42,1	54,5	28,6	43,5	0	0	20,0	50,0	60,0
**Discussion**													
Limitations	Trial limitations (potential bias, imprecision)	**20**	82,4[50,0–100]	96,0	94,7	90,9	71,4	73,9	100	50,0	100	50,0	66,7
Generalisability	Generalisability (external validity, applicability) of findings	**21**	75,6[46,7–100]	88,0	63,2	90,9	57,1	95,7	100	100	60,0	50,0	46,7
Interpretation	Interpretation consistent with results, balancing benefits and harms, and considering other relevant evidence	**22**	100[100–100]	100	100	100	100	100	100	100	100	100	100
**Other info.**													
Registration	Registration number and name of trial registry	**23**	50,0[0–100]	68,0	57,9	36,4	85,7	100	0	50,0	40,0	50,0	6,7
Protocol	Where the full trial protocol can be accessed	**24**	0[0–9,1]	0	0	9,1	7,1	4,3	0	0	0	0	6,7
Funding	Sources of funding and other support, role of funders	**25**	97,9[0–100]	100	94,7	81,8	100	95,7	100	100	100	0	40,0

Table 2 shows 119 randomised controlled trials published between January and December 2011. n, number of RCTs included per journal. Total values of all included journals and per journal show the percentage of adherence to the CONSORT Checklist items in median and [range]. All other values are presented in [%] of reported items per all RCTs belonging to each journal. The denominator of the item 11b is shown for each analysis behind the value, since not all RCTs were blinded. The item description is an abbreviated form, derived from the CONSORT statement 2010. AJRCCM: American Journal of Respiratory and Critical Care Medicine

### Other analyses

**Fig 4:**
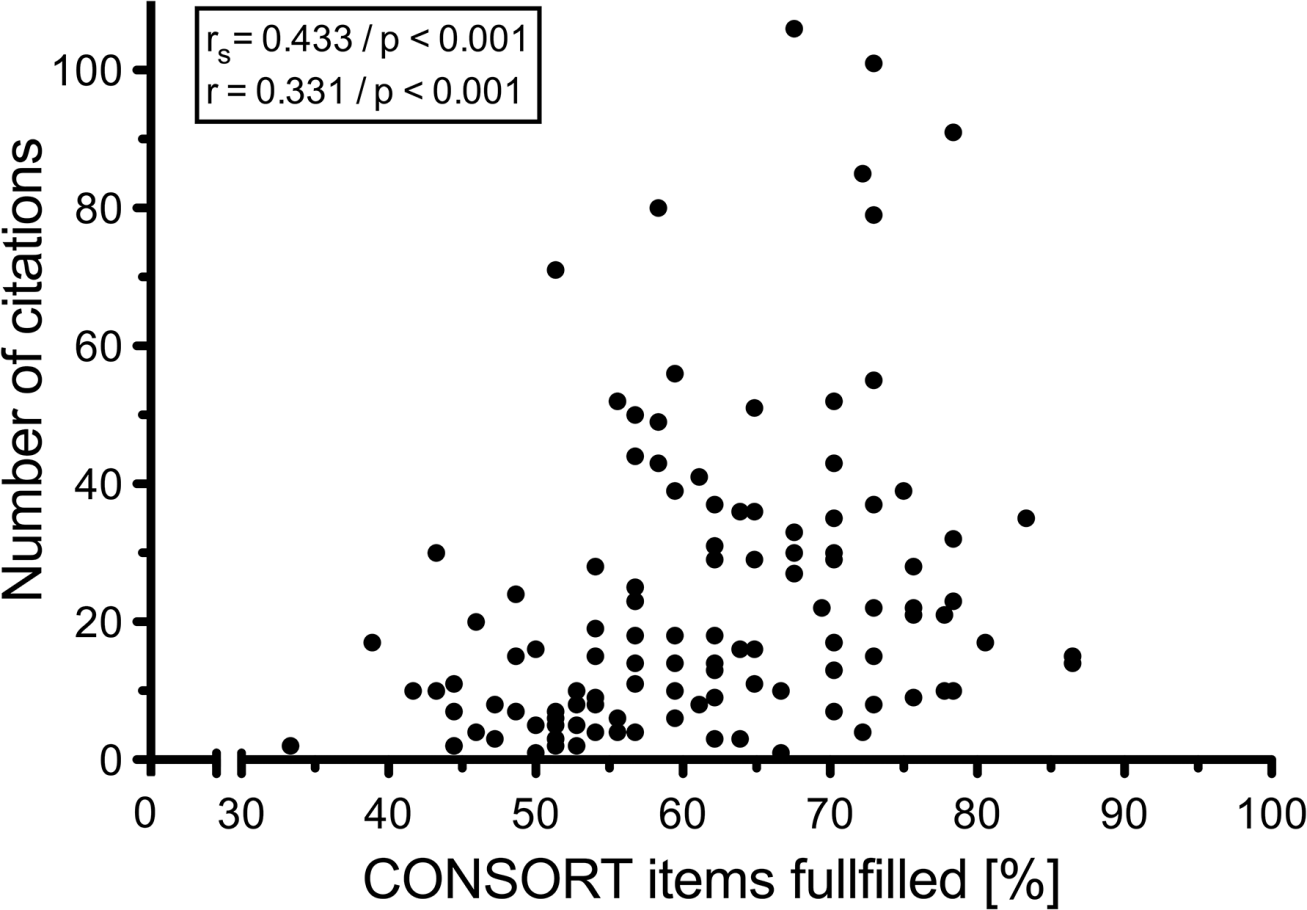
Correlation of RCT adherence to CONSORT checklist and citation frequency. Correlation of citation frequency from 01.01.2012–31.12.2014 and fulfilled CONSORT items of each RCT.

The median citation frequency was 17 with a range from 1 to 106 citations, the Huber`s M-estimate was 18. We found a significant correlation between the percentage of reported items per RCT and the citation frequency in 2012–2014 (r_s_ = 0,433; p<0,001, r = 0,331; p<0,001) (Fig 4). The further correlation analysis between the percentage adherence to the CONSORT items per article and the respective impact factor of the journal in the year 2011 showed a significant correlation with r_s_ = 0,386; p<0,001. The overview of the included journals and important properties are shown in Table 3.

**Table 3:** Journals’ information.

Journal	IF 2011	Number of articles included (n)	Number of citations
American Journal of Respiratory and Critical Care Medicine	11,080	25	1101
Critical Care Medicine	6,330	19	455
Intensive Care Medicine	5,339	11	260
CHEST	5,250	14	241
Critical Care	4,607	23	424
Journal of Neurotrauma	3,654	1	8
Resuscitation	3,601	4	122
Pediatric Critical Care Medicine	3,129	5	36
Shock	2,848	2	22
Minerva Anestesiologica	2,656	15	69

List of included journals, journals´ s impact factor, number of published RCTs in each journal and total number of citations assessed form 01.01.2012–31.12.2014 of the analysed RCTs per journal.

A 10% crosscheck sample of RCTs revealed a high inter-rater reproducibility with kappa = 0,925 (n = 444).

## Discussion

### Key Results

1. The adherence to the current CONSORT statement of RCTs published 2011 in the "top-ten" journals belonging to the category of critical care medicine was only 61,1% (median), with a range of 33,3 to 86,5% per RCT.
2. Even essential CONSORT items were only poorly reported. For example, the sample size calculation was reported in only 39,5% of 119 analysed RCTs.
3. The adherence to the CONSORT statement should be enforced on submission of articles to journals.

### Interpretation

We evaluated the reporting-adherence to each item of the current CONSORT checklist [9], after the latest revision of the CONSORT statement in 2010. This analysis was restricted to all RCTs published 2011 in the top ten journals in the category of critical care medicine, identified by the Thomson Reuters journal citation report (via ISI Web of Knowledge). One previous review analysed papers restricted to one of the top ten journals (Intensive Care Medicine) and evaluated only three items of the CONSORT checklist [19]. They included RCTs until 2010 and there was no improvement of reporting quality after the former revision of the CONSORT statement 2001 [7].

Some items of the CONSORT Checklist are mandatory for a methodological high quality RCT. These include, in our opinion, predefined objectives and hypotheses (2b), clear eligibility criteria (4a), pre-specified outcome parameter (6a), a sample size determination (7a), allocation concealment (9), blinding (11a), methods used for statistical analysis (12a), flow chart (13a), results for each outcome (17a), interpretation of the results (22) and limitations of the study (20). Considering these items among the other CONSORT Checklist items before launching a trial may help to improve the design, conduction and analysis of a trial [2].

In this retrospective analysis we revealed that in total, only slightly more than half (median and M-estimate of 61,1%) of the required CONSORT checklist items were reported, with a range of 33,3 to 86,5% per RCT. Our results underline recent results of three RCT-reporting-quality analyses, which also analysed the adherence to all CONSORT items of the newest CONSORT Checklist. Elia et al. showed in total 41% adherence of trials published 2010 in the European Journal of Anaesthesiology [20], Ahmadzadeh et al. identified 74% of reported items in five high impact factor medical journals in 2011 and 2012 [21] and Münter et al. revealed 60% adherence in the top ten ranked anaesthesiology journals in 2011 [22].

We identified only two items (2a and 22), which were reported in every RCT. These items are essential for the performance of RCTs. In contrast only 55,5% of the RCTs were marked as “randomised” in the title, although it is known that literature search is frequently performed by screening the titles. However, 55.5% is much higher than the rate of 24–33% reported in previous assessments of the literature [10,19,20]. In contrast, pre-specified outcome parameters (6a) were reported highly (96,6%). Previous analyses again identified lower reporting rates; only 53% of the RCTs reporting item 6a [10] and Elia et al. discovered a frequency of 48% [20]. Our previous analysis of the top ten journals in the category of anaesthesiology (2011) showed a reporting adherence to 6a of only 72% [22]. Exaggerated estimates of the intervention benefit are likely in RCTs with inadequate conduction and reporting of blinding, sequence generation and allocation concealment [5,23]. Nevertheless, these items were rarely reported in our analysis (48,7%, 61,3% and 37% respectively). All three items show a widespread variety in other reporting-quality analyses (25–88%) [10,19,21,24,25].

A sample size calculation (item 7a) should be performed for every RCT beforehand to avoid ethically unnecessary exposure of participants in under powered studies [8,26]. Omitting reporting of sample size calculation hinders the readers from verifying the results of the trial. It is alarming, that this item was only reported in 39,5%, even lower than in the analyses of Latronico (43%) and Hopewell (44%) [10,19]. It seems that item 7a, 8a, 9 and 11a are significantly more frequently reported in RCTs published in general medicine journals with very high impact factors. A sample size calculation (item 7a) was reported in 82,6% of the analysed RCTs by Mills et al. and 62% in the analysis of Ahmadzadeh et al. [21,24]. It was assumed that the amount of reported CONSORT items is more frequent in general medicine journals than in specialty journals [10,24]. Charles et al. investigated the reporting frequency and the accuracy of the sample size calculation in RCTs published in six “high impact” medical journals [26]. They also found a much higher reporting frequency of this item 7a (95%). Of note there was a high discrepancy between the reporting frequency and the quality of the reported items for the sample size calculation. Only 34% reported all data, which enabled an accurate recalculation and showed correct assumptions for the sample size calculation. Interestingly, the recently published analysis of top ten anaesthesiology journals presented an 85% adherence to item 7a [22]. Similar to the analysis of Elia et al. [20], changes to methods (item 3b) and outcomes (item 6b) were reported less than 1%. Furthermore the reason for the trial termination (item 14b) was rarely (19,3%) reported in the results part of the most RCTs. In our opinion a lack of protocol changes should be reported for clarity. Similarly the reason for trial termination should be reported in each RCT. It cannot be excluded, that some researchers misinterpreted these items and thought to report them only, if there were some unexpected protocol deviations. Trial registration becomes more and more important for RCTs, as it may reduce publication bias and reveal changes to the pre-specified primary outcome variables of the trials [27]. Our analyses showed that 61,3% of the RCTs were registered. This is obviously more than in the analysis of Hopewell et al. (9%) [10], but lower compared to Ahmadzadeh et al. (76%) [21]. Of note, high quality of reporting does not exclude a trial conducted with strong bias [28]. Furthermore the lack of report in the methods does not always mean inadequate methodology [29].

It remains unclear why no article has reported more than 90% of the CONSORT items. This raises the question, whether the CONSORT statement requires a too high reporting standard or if CONSORT is consciously neglected aiming to conceal trial's inadequacies [9]. Nevertheless, CONSORT addresses minimum criteria that were established evidence based by the CONSORT Group [9]. According to other analyses [10,16,19–22], we recommend the strictly adherence to the CONSORT statement for every RCT in the future. Even, if the journals have limitations for the maximum of used words in the article, the CONSORT items should be addressed at least in supplemental data. This would minimize reporting of biased results [2] and enable an easier comparison of RCTs for the readers. A mandatory totally completed CONSORT checklist at the submission process and an endorsement by funding agencies, would facilitate achieving this aim.

### Limitations

Our study has some limitations. First, the analysis was restricted to the year 2011. We decided to analyse that year, as the latest CONSORT statement revision was published in March 2010 [8,9]. This selection might have introduced the risk of overlapping between the publication of the latest CONSORT revision and the manuscripts, published early in 2011, but submitted before March 2010. Of note, the cornerstones of the CONSORT statement existed already since twelve years and the most items of the current checklist were already present since their revision in 2001 [7]. Hence we cannot exclude that the reporting quality in the field of critical care medicine has not already improved since 2012. Further investigations are required to continually appraise whether there is a trend to improvement of adherence to the CONSORT statement in critical care medicine articles. A second limitation is our journal selection. To our opinion, it was the most objective way to choose them from the ISI Web of Knowledge as described by Altman [30]. There were 26 journals indexed in the category of critical care medicine in 2011, and we decided to make the cut off after the top-ten journals, for feasibility reasons. Including the journals according to their impact factor may have induced a selection bias and does not exclude that critical care medical journals with lower impact factors provide the same or different reporting quality [26]. Furthermore the amount of RCTs per journal was unequally distributed and the Journal of Neurotrauma and the journal Shock have published significantly less RCTs than the other journals. This has to be taken in account when considering our overall result of this analysis. Another limitation is, that we did not contact the authors to obtain the study protocols or information about any unplanned changes during the trial conduction or analyses. Only one trial had published their study protocol online available. Therefore we have assigned non-adherence for the respective items if changes were not reported in the articles.

## Conclusions

We revealed, in the top-ten impact factor weighted journals of critical care medicine, a poor median proportion of 61,1% reported CONSORT items per RCT with a range from 33,3–86,5%. Further investigations reviewing reporting quality improvement in the category of critical care medicine are absolutely required.

## Supporting Information

S1 Checklist STROBE checklist. (DOCX)

S1 Table Defaults for assigning the CONSORT checklist items. (DOCX)
