# Supplementary material for: CONSORT Item Reporting Quality in the Top Ten Ranked Journals of Critical Care Medicine in 2011: A Retrospective Analysis
Source: PLoS One. 2015 May 28;10(5):e0128061. doi: 10.1371/journal.pone.0128061 (PMC4447424; doi:10.1371/journal.pone.0128061)
Supplement: S1 Table — (DOCX) [file pone.0128061.s002.docx]

**Additional file 1. Defaults for assigning the items**

|  | **Item n°** | **Yes** | **No** | | | | **n.a.** | |
| --- | --- | --- | --- | --- | --- | --- | --- | --- |
| **Titel & abstract** | **1a** | “Randomised trial” is explicitly stated in the title. | “Randomised trial” is **not** explicitly stated in the title. | | | | - | |
|  | **1b** | Structured abstract is available. | Structured abstract is **not** available. | | | | - | |
| **Introduction** | **2a** | Scientific background and rationale are explained in the introduction. | Scientific background and rationale are **not** explained at all, **or** are explained in another part of the manuscript than the introduction. | | | | - | |
|  | **2b** | Specific objectives or hypotheses are stated in the introduction. | Specific objectives or hypotheses are **not** stated at all, **or** not stated in the introduction. | | | | - | |
| **Methods**  Trial design | **3a** | Exact description of the trial design is given and in case of drug trials the phase of the trial (I-IV) is described. | Trial design and trial phase of drug trials is **not** provided, **or** only mentioned in the abstract but not in the Methods. | | | | - | |
|  | **3b** | Method changes after trial commencement are clearly described. | Method changes after trial commencements are **not** described, although the trial has obviously some.  Method changes are not reported and as we did not analyse the original study-protocol we cannot prove that this item is not applicable for the respective trial, therefore: non-adherent | | | | - | |
| Participants | **4a** | Participant eligibility criteria are stated clearly. | Participant eligibility criteria are **not** stated clearly. | | | | - | |
|  | **4b** | Clear statement of the setting and location.  In case the methods refer to “our hospital” or the trial is called “multicentre” the hospital/care provider or the participating centres have to be mentioned in detail. | In case there was **no** statement of setting and surrounding this criterion has to be assessed as not fulfilled. | | | | - | |
| Interventions | **5** | Exact description of the intervention for the study- / control-group. When using drugs, the ways of application, the name of the drug as well as the time and dose have to be named. | Intervention **not** described in detail. | | | | - | |
| Outcome | **6a** | Exact definition of primary and secondary outcome measures. Additionally it has to be defined when and how the outcome measurers were assessed. | **Not** exact predefined outcome measures. | | | | - | |
|  | **6b** | In case of any deviations from the trial protocol, regarding the outcome measures, these deviations have to be reported. | Identified deviations of outcome measures in the paper, without naming the reasons in the methods part.  Item not reported anywhere: as we did not analyse the original study-protocol, we cannot prove that this item is not applicable for the respective trial, therefore item is non-adherent. | | | | - | |
| Sample size | **7a** | Detailed sample size calculation was performed. It is clearly stated how and with which program the calculation was conducted. | In case the detailed calculation in the method part was carried out without naming the program the criterion is not fulfilled. | | | | - | |
|  | **7b** | Interim analyses and stopping guidelines are reported. | Interim analyses were conducted, as mentioned at any other part of the paper, but they are not stated explicitly in the methods part. Item not reported anywhere in the paper: as we do not analyse the original study-protocol, we cannot prove that this item is not applicable for the respective trial, therefore item has to be counted “non-adherent”. | | | | - | |
| Random sequence generation | **8a** | Generation of the random allocation sequence is clearly stated in Methods part. | Generation of the random allocation sequence is **not** clearly stated in the Methods part, or is only stated somewhere else in the paper. | | | | - | |
|  | **8b** | Type of randomisation is described with all restrictions in detail. Any restriction has to be listed. | Type of randomisation is **not** described. | | | | - | |
| Allocation concealment | **9** | Allocation concealment described in detail. | Allocation concealment **not** described in detail. | | | | - | |
| Implementation | **10** | All 3 steps of randomising participants into the trial are described. | In case these steps are **not** named and described in detail the criterion is not fulfilled. | | | | - | |
| Blinding | **11a** | Description who was blinded in detail. Each blinding has to be described in details as it is not sufficient to mention e.g.“double blind” as a precise definition does not exist. Without blinding it has to be reported, that blinding was not performed. | Blinding is **not** described in detail. Blinding is described insufficient e.g. “double blind” as a precise definition does not exist. Trial was not blinded and this kind of sentence is **not** reported anywhere in the text: “blinding was not performed” | | | | - | |
|  | **11b** | Similarities of the intervention, to assure blinding of participants/healthcare providers are reported. | Similarities of the intervention, to assure blinding of participants / healthcare providers are **not** reported. | | | | Similarity of interventions not necessary/relevant for this trial, as it was not blinded. | |
| Statistical methods | **12a** | The statistical methods are mentioned exactly for each analysis. Only one dataset per participant is evaluated. | Not every statistical method, for each reported result is stated. | | | | - | |
|  | **12b** | Description of the methods for any additional analyses is stated | Additional analyses were performed, but not described in detail. Additional analyses are not reported: as we do not access the original study-protocol, we cannot prove that this item is not applicable for this trial, therefore it has to be allocated “non-adherent”. | | | | - | |
| **Results**  Participant Flow | **13a** | Correct presentation of the study in the participant flow. (Number of included, randomised and analysed patients in each group) | **No** participant flow.  Incorrect/insufficient participant flow. | | | | - | |
|  | **13b** | For each group, all reasons of exclusions after randomisation are stated. | Exclusions are **not** mentioned in the flow chart or they are **not** sufficiently described. | | | | - | |
| Recruitment | **14a** | The period of recruitment and follow up is clear stated. | The recruitment period and follow up time are **not** described exactly, e.g. only the year is reported or it is mentioned at any other part of the paper. | | | | - | |
|  | **14b** | It is clearly described, why the trial ended in the result or methods part of the paper. | It is nowhere reported, why the trial ended. | | | | - | |
| Baseline data | **15** | A table showing the baseline demographical and clinical characteristics of each group is present. | A table showing the baseline demographical and clinical characteristics of each group is **not** present. | | | | - | |
| Number analysed | **16** | The number of participants, included in each analysis, is reported.  It is stated which kind of data-analysis regarding the number of randomised participants is used. (Intention-to-treat vs. per protocol/on-treatment) | It is **not** reported, which patients were included/excluded from the analysis and which kind of analysis is performed. | | | | - | |
| Outcome & estimation | **17a** | The estimated effect size and its precision of all pre-specified outcomes are reported for each group. Only p-value reporting is insufficient. | The estimated effect size and its precision of all predefined outcomes are **not** accurately reported for each group. | | | | - | |
|  | **17b** | Within binary outcomes the absolute and relative effect size are reported. | For binary outcomes the absolute and relative effect-size are **not** or **not both** reported. | | | | - | |
| Ancillary analyses | **18** | If additional analyses were performed, it has to be mentioned, if they were pre-specified or subsequent analyses and why they were performed. | Details regarding the reason for performed additional analyses are missing.  No additional analyses are reported: as we do not access the original study-protocol, we cannot prove that this item is not applicable for this trial, therefore it has to be allocated “non-adherent”. | | | | - | |
| Harms | **19** | The frequency of adverse events (including the absence of them) per group is reported. | The frequency of adverse events per group is **not** reported or the absence of adverse events is **not** reported. | | | | - | |
| **Discussion**  Limitations | **20** | All study limitations (also absent) are clearly reported. | Limitations are not reported, or it is not explicitly stated that there are no limitations. | | | | - | |
| Generalisability | **21** | It is clearly stated, if the study results can be generalised to other sites / patients / populations, different from those enrolled in the trial.  It is stated, if the results are applicable to other drugs / doses of drugs / timing / administration route of drugs.  It is stated, that the results are only applicable to their study-circumstances and that further trials are needed to proof their results. | It is nowhere mentioned, if the results are valid for other circumstances. | | | | - | |
| Interpretation | **22** | A brief review of the present literature of the examined question is given and a relation to the results of the present paper is made. | The results of the paper are not related to the results of present literature. | | | | - | |
| **Other information** |  |  |  |  |  |  |  |  |
| Registration | **23** | Registration number and name of trial registry are provided, or reasons for not performed registration are explicitly stated. | Registration number and name of trial registry are **not** provided, or **no** reasons for not registering are explicitly given. | | | | - | |
| Protocol | **24** | It is explicitly reported, where the complete trial protocol can be accessed. | The accessibility of the complete trial protocol is **not** explicitly stated. | | | | - | |
| Funding | **25** | Financial funding and support is clearly reported. In case of drug studies, the supply of the drug is specified. | Financial funding and support is **not** clearly reported. The supply of drugs in drug studies is **not** specified. | | | | - | |
